# Preloaded vs manually loaded IOL delivery systems in cataract surgery in the largest ambulatory surgery center of northwestern China: an efficiency analysis

**DOI:** 10.1186/s12886-020-01721-5

**Published:** 2020-12-01

**Authors:** Yazhen Wu, Hong Yan, Weijia Yan

**Affiliations:** 1 Xi’an Fourth Hospital, Shaanxi Eye Hospital, Affiliated Xi’an Fourth Hospital, Northwestern Polytechnical University, Xi’an, 710004 Shaanxi Province China; 2 Department of Ophthalmology, University of Heidelberg, 69120 Heidelberg, Germany

**Keywords:** Preloaded intraocular lens, Cataract, Cataract throughput, Hospital revenue

## Abstract

**Background:**

To compare the efficiency of preloaded vs manually loaded IOL (P-IOL vs M-IOL, respectively) delivery systems in cataract surgery in the largest ambulatory surgery center of Northwestern China.

**Methods:**

A total of 200 cases were included in this prospective, observational study. Time and motion data were collected in a one- or two-operating room (1-OR or 2-OR, respectively) scenario. A model of the efficiency and revenue implications of introducing a preloaded delivery system for IOLs in cataract surgery was used to estimate the changes in cataract throughput and hospital revenue through transitioning from the M-IOL delivery system to the P-IOL system.

**Results:**

In the 1-OR scenario, the mean total case time was 16.9 min using P-IOL, which was a 7.7% reduction compared with M-IOL (*P* < 0.01). In the 2-OR scenario, the mean total surgeon time was 10.8 min using P-IOL, which was a 7.8% reduction compared with M-IOL (*P* < 0.05). By switching from M-IOL to P-IOL, annual throughput will increase by 5.2% (960 cases) in the 1-OR scenario and 7.7% (1440 cases) in the 2-OR scenario, accompany by an increase in revenue of approx. 284,352 USD in the 1-OR scenario and approx. 426,528 USD in the 2-OR scenario.

**Conclusion:**

This report is the first of a comparison of two IOL delivery systems in China using different settings in the scenario. IOL delivery with preloaded systems is time saving in both the 1-OR scenario and the 2-OR scenario. Moreover, switching from the M-IOL delivery system to the P-IOL system holds potential to increase cataract throughput and hospital revenue.

## Background

With the development of surgical instruments and surgical skills, cataracts can be easily, safely and cost-efficiently treated with a routine operation [1, 2]. However, cataracts remain the second leading cause of visual impairment and the leading cause of vision loss throughout the world [3–5]. A recent study showed that, in 2010, cataracts resulted in 94 million people being visually impaired and 20 million blind [6]. By 2020, more than 30 million people are predicted to undergo cataract surgery annually worldwide [7].

As the population ages, the prevalence of cataracts is expected to increase correspondingly. In China, among people aged 45–89 years old, the number of cataract cases was 50.75 million in 1990 and 111.74 million in 2015 [3]. By 2050, it is predicted that 240.83 million people aged 45–89 years old will be affected by cataracts [3]. The doubling of cataract patients and the delay in cataract surgeries pose challenges for limited healthcare resources and governments. More efficient and effective surgical techniques are needed to provide reliable outcomes and to improve cataract throughput.

In standard cataract surgery, the opacified lens is phacoemulsified, and an artificial foldable intraocular lens (IOL) is inserted through a small incision [8, 9]. The IOL is manually folded using forceps and is inserted into an injector system. Loading of the IOL requires more extended time for the overall surgery and longer training for the operating staff, which is time-consuming because of the differences in the designs of the manual injection systems from various companies. Furthermore, manual loading of the IOL increases the risk of damage to it, as well as surgeon error in holding and folding of the IOL [10, 11]. To address these concerns, preloaded IOLs (P-IOLs) have been developed, holding the potential to reduce IOL loading errors, eliminate additional handling of IOLs, decrease total case time, and improve throughput [10, 11].

To evaluate the efficiency of P-IOL vs manually loaded IOL (M-IOLs) delivery systems in cataract surgery, a prospective, observational study was conducted. We compared the surgery time between the P-IOL and M-IOL delivery systems in one- and two-operating room scenario. Using the efficiency and revenue implications of introducing a preloaded delivery system for IOLs in cataract surgery systems, we further estimated the potential throughput and hospital revenue using P-IOL without more staff.

## Methods

### Study participants

All of the studies were approved by the Xi’an Fourth Hospital Ethics Committee and were performed following the ethical standards. We state that they adhered to the tenets of the Declaration of Helsinki. In the present study, there were no changes to patient care (e.g., patients received standard of care), and patients chose the IOL according to their preference; we received written informed consent for the surgeries from all of the patients. Given that time recording is a routine part of surgeries, the Xi’an Fourth Hospital Ethics Committee waived the need for additional patient written informed consent.

Xi’an Fourth Hospital is a tertiary referral center in Xi’an, China. There are four operating rooms in the ambulatory surgery center of Xi’an Fourth Hospital. In 2019, 18,635 cataract surgeries were performed in our center. The number of scheduled cataract surgeries is 5 d/wk. The surgeries were performed 48 wks per year, excluding 4 wks of legal holidays. All of the cases were routine cataract surgeries, with 18,069 (97.0%) being monofocal IOL insertions. We conducted a prospective, observational study. Patients who visited Xi’an Fourth Hospital from September 2019 to December 2019 were included. The other inclusion criteria were age ≥ 45 years old, with a nuclear or cortical cataract grade 2 to 5 according to the Lens Opacities Classification System, version III (LOCS III), and undergoing routine cataract surgery in a single procedure. The exclusion criteria included corneal pathology, previous ocular surgery, preexisting corneal astigmatism greater than 2.00 diopters, a postoperative visual acuity target other than emmetropia, wearing of contact lenses during the preceding 6 months, and previous eye trauma.

The P-IOL system was utilized in our hospital before the beginning of this study. The surgeon already had experience using the P-IOL or M-IOL systems for routine cataract surgeries. For all of the cases, the surgeon loaded the M-IOL himself. The loading process included preparing the ophthalmic viscosurgical device-filled cartridge and advancing the plunger to place the lens near the cartridge tip.

### Data collection

Time and motion data were collected over a total of 12 surgery days as described previously [10]. The time of the procedures was recorded using a stopwatch. The TECNIS iTec Preloaded IOL (P-IOL, PCB00, Abbott Medical Optics Inc., Santa Ana, CA, USA) and the TECNIS 1-Piece IOL (M-IOL, ZCB00, Abbott Medical Optics Inc.) were used throughout the study.

The operating setup time was defined as the time between the opening of the ophthalmology pack and preparation of all of the surgical items (including preparation of instruments in the sterile field, sterilization of the surgical field and application of the surgical drape). Surgery began when the surgeon positioned the microscope and provided a verbal indication that surgery had begun. The surgery ended when the surgeon finished checking the status of the eye and provided a verbal indication that the surgery was complete. Surgeon lens time was defined as the length of time that the surgeon spent loading, inserting, and positioning the lens in the eye. Tear-down time was defined as the time following surgery until the nurse finished applying disinfectant to the surgical table surfaces. The total case time included the operating setup, surgery, and tear down times.

### Model structure

A model structure was used to estimate the change in cataract throughput and revenue by adoption of a P-IOL system. The revenue were converted to US dollars (exchange rate 1 USD = 7.09 CNY; 15 October 2019). In our ambulatory surgery center, the average cost of cataract surgery is 2100 CNY (296.2 USD).

#### OR scenario

Within a 1-OR scenario, the total time savings per surgery day was calculated. If the total case time savings per surgery day exceeded the mean time between the start of the operating setup and the completion of the operating room’s tear down with the P-IOL system, then an additional case, or cases, could be added to the surgery day without additional staffing.

#### OR scenario

In a 2-OR scenario, if the total surgeon time savings per surgery day exceeded the total surgery time with the P-IOL system, then an additional case, or cases, could be added to the surgery day without additional staffing.

### Statistical analysis

All of the data were presented as the means ± standard deviations (SDs). The Shapiro-Wilk test confirmed the normal distribution of the data. Statistical comparisons were made by the *t*-test (SPSS23.0, IBM, Armonk, NY, USA). A statistical significance level of 0.05 was applied.

## Results

In the present study, all of the cataract surgeries were performed by one experienced surgeon. Before starting the study, the P-IOL system (TECNIS iTec Preloaded Delivery System) had been adopted in our hospital for more than 2 years. Thus, the surgeon was familiar with both preloaded IOL and M-IOL delivery systems. A total of 200 routine cataract cases were enrolled in the study, of which 98 were performed with P-IOL, and 102 were performed with M-IOL. Details of the study population can be seen in Table 1.

**Table 1:** Characteristics of the study population

Procedure		1-OR		2-OR	
		M-IOL	P-IOL	M-IOL	P-IOL
Case Number		47	51	54	48
Gender	Male	23	24	26	24
	Female	24	27	28	24
Age (Years)	Average	63.2	60.8	65.3	63.6
	Range	45.5–85.8	46.3–88.1	45.1–83.3	45.2–90.2

Time and motion data are shown in Tables 2 and 3, respectively. In the 1-OR scenario (Table 2), the setup time, lens time and tear down time were significantly reduced using P-IOL compared with M-IOL (*P* < 0.01). There was a reduction trend in surgery time using P-IOL (− 6.8% vs M-IOL, *P* > 0.05), but it did not reach statistical significance. The mean total case time was 16.9 min using P-IOL, which was a 7.7% reduction (1.1 min per case) compared with M-IOL (*P* < 0.05). In the 2-OR scenario (Table 3), the mean total surgeon time was 10.8 min using P-IOL, which was a 7.8% reduction (0.9 min per case) compared with M-IOL (*P* < 0.05).

**Table 2:** Time and motion data in the 1-OR scenario

Procedure	1-OR			
	M-IOL (m)	P-IOL (m)	Diff (%)	*P*-value
Setup time	4.2	3.8	−9.5	< 0.01
Surgery time	11.8	11.0	−6.8	> 0.05
Lens time	1.3	0.7	− 46.2	< 0.01
Teardown time	2.3	2.1	−8.0	< 0.01
Total case time	18.2	16.9	− 7.7	< 0.01

**Table 3:** Time and motion data in the 2-OR scenario

Procedure	2-OR			
	M-IOL (m)	P-IOL (m)	Diff (%)	*P*-value
Surgery time	11.7	10.8	−7.8	< 0.05
Lens time	1.3	0.7	−46.2	< 0.01
Total case time	11.7	10.8	−7.8	< 0.05

**Fig. 1:**
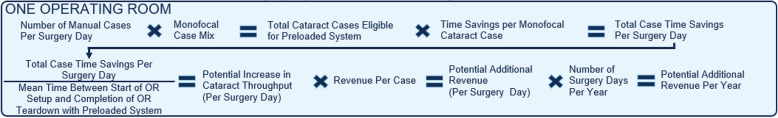
The model structure of 1-OR

**Fig. 2:**
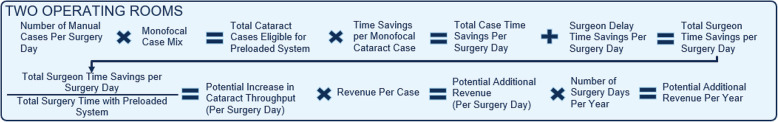
The model structure of 2-OR

To estimate the change in cataracts throughput and hospital revenue from switching from the M-IOL delivery system to the P-IOL system, efficiency and revenue implications of introducing a preloaded delivery system for IOLs in cataract surgery model were used (as shown Figs. 1 and 2, respectively). In 2019, 18,635 routine cataract surgeries were performed (77.6 per surgery day) in our ambulatory surgery centers, with 97.0% being monofocal IOL insertions. Assuming that the center runs as four independent 1-OR scenarios, 4 more operations will be performed per surgery day (Table 4). The annual throughput will increase by 5.2% (960 cases), accompanied by an increase in revenue by 284,352 USD (Table 5). Assuming that the center runs as 2 independent 2-OR scenarios, 6 more operations will be performed per surgery day (Table 4). The annual throughput will increase by 7.7% (1440 cases), accompanied by an increase in revenue by 426,528 USD (Table 5).

**Table 4:** Impact of P-IOL efficiencies over the entire surgery day in the 1-OR or 2-OR scenario

Scenario	1-OR	2-OR
Mean number of M-IOL cases/surgery day	18.8	37.6
Monofocal case mix (%)	97.0	97.0
Mean total case time saving/case	1.4 min	0.9 min
Total case time savings/surgery day	26.4 min	33.9 min
Potential increase in cataract cases/surgery day	1.6	3.1
Potential increase in cataract cases/surgery day (Our ambulatory surgery center runs 4 operating rooms)	4	6

**Table 5:** Estimation of annual cataract throughput and revenue increase by applying P-IOL in the ambulatory surgery center running 4 operating rooms

Procedure	1-OR		2-OR	
	M-IOL	P-IOL	M-IOL	P-IOL
Surgery days/year	240	240	240	240
Mean cases/surgery day	77.6	81.6	77.6	83.6
Total cases/year	18,635	19,595	18,635	20,075
Additional annual throughput	960 (5.2%)		1440 (7.7%)	
Additional annual revenue (USD)	284,352		426,528	

## Discussion

With the accelerated aging of the population, the increasing number of cataract patients and delays in cataract surgery pose challenges for limited healthcare resources and governments. Thus, more efficient and effective surgical techniques are needed to provide reliable outcomes and improve cataract throughput. In the present study, we compared the efficiency of P-IOL vs M-IOL in our ambulatory surgery center. We found that application of the P-IOL significantly reduced the total surgery time both in the 1-OR and 2-OR scenarios. Using our designed model, it was estimated that the switch from M-IOL to P-IOL could increase the throughput by 5.2–7.7% and increase the revenue by 284,352 to 426,528 USD without additional staffing and operating rooms.

Years ago, nearly all cataract surgeries required inpatient hospitalization. Recently, an increasing number of cataract surgeries have been performed at ambulatory surgery centers in China, improving cataract throughput and convenience for patients and surgeons. In addition, performing cataract surgeries at ambulatory surgery centers enhances surgeon productivity, decreasing out-of-pocket patient costs, and per-case costs for insurers [12]. In 2015, an ambulatory surgery center was established in our hospital to perform routine cataract surgeries, substantially improving cataract throughput. With the acceleration of aging of the population in China and worldwide, the cataract patients will double in 30 years, posing challenges for healthcare systems and limited healthcare resources. For routine cataract surgery, the average surgery time is as short as 10–20 min. Therefore, even small changes in the efficiencies of operating processes and surgical techniques can quickly add to meaningful differences in cataract throughput and efficiency of the healthcare system.

To provide better operating conditions, P-IOL delivery systems were developed. The perceived benefits of P-IOL delivery systems include the elimination of IOL injector loading variability, avoidance of potential IOL loading errors, lower risk for instrument contamination and reduced operation time [10, 11, 13–15]. In this study, our data showed that, in a 1-OR scenario, application of the P-IOL system reduced the average total case time by 1.3 min (− 7.7%) relative to the M-IOL system. The reduction of the total case time was largely the result of the reductions in operating setup time, tear down time, surgery time and IOL delivery time. The reductions in operating setup time might be ascribed to that the injector in the M-IOL group is reusable, and more time is needed to confirm the sterilization of the injector, open the sterilized package and check whether the injector is normal. In a 2-OR scenario, application of the P-IOL system reduced average total case time by 0.9 min (− 7.8%) relative to the M-IOL system. The reduction of the total case time was the result of the reductions in surgery time and IOL delivery time, rather than surgery delay time. This outcome was somewhat different from previous findings that the reduction of surgery delay time also contributed to the reduction of total cases [10], which might be ascribed to the staff structure optimization in our institution, according to which a circulating nurse and a resident were in one operating room. Thus, they cooperated well and had sufficient time to prepare the surgery equipment and patients.

The staff and operating room are both valuable and expensive. Maximization of staff and operation room efficiency could provide medical serves to more patients. In this study, the efficiency and revenue implications of introducing a preloaded delivery system for an IOL model were used to evaluate the changes in cataract throughput and hospital revenue from switching from the M-IOL delivery system to the P-IOL system. Within a 1-OR center, if the total case time savings per surgery day exceeds the mean time between the start of the operating setup time and the completion of the operating’s tear down with the preloaded system, then an additional case, or cases, can be added to the surgery day without additional staffing. In a 2-OR center, if the total surgeon time savings per surgery day exceeds the total surgery time with the preloaded system, then an additional case, or cases, can be added to the surgery day without additional staffing. Thus, by switching from the M-IOL system to the P-IOL system, annual throughput will increase by 5.2% (960 cases) in the 1-OR scenario and 7.7% (1440 cases) in the 2-OR scenario. Similarly, Jones, et al. reported that, in a single-OR cataract surgery center in Canada, the cataract throughput increased by 9.9% (36 cases per year) by switching from the M-IOL system to the P-IOL system without increasing staff or surgeon capacity. In 2-OR cataract surgery centers (France and the USA), the cataract throughput will increase by 5.7% (90 per year in France) and 4.0% (96 per year in the USA), respectively.

The cost of cataract surgery per case varies widely depending on location and setting in which the surgery is performed. A study of nine European countries showed that the cost of cataract surgery per case ranged from 344 to 1177 USD. In our ambulatory surgery center, the average cost of cataract surgery is 296.2 USD, which means 296.2 USD of revenue for the hospital. Switching from the M-IOL delivery system to the P-IOL system, the hospital could increase its revenue by 284,352 USD in the 1-OR scenario and 426,528 USD in the 2-OR scenario. In contrast, for hospitals like our ambulatory surgery center, for which the salaries for the surgeon and staff depend on the throughput, translating from the M-IOL delivery system to the preloaded system could potentially increase the input of the surgeons and staff.

Although the findings are promising, the current study had several limitations. First, the present study was limited by the data collection being performed at a single ambulatory surgery center by a single observer over a short period of time, thereby limiting the generalizability of the findings. Second, in the present study, the surgeon loaded the M-IOL himself, while in hospitals in which the staff helps to load the M-IOL, the surgery time is expected to be further reduced. Third, for feasibility reasons, only one type of IOL was compared in this study. In the future, a more detailed record of the different types of IOL from various manufactories would provide better data for decision making. Fourth, the present study focused on the influence on cataract throughput and revenue by the application of P-IOL, and we did not specifically track patient outcomes before and after implementation of P-IOL or M-IOL. The surgeon did not anecdotally note evidence of increased intraoperative or postoperative complications.

## Conclusions

This report is the first of a comparison of two IOL delivery systems in China using different settings of the scenario. Our study showed that using the P-IOL delivery system for routine cataract surgeries reduces total case time relative to the M-IOL delivery system in both the 1-OR scenario and 2-OR scenarios. The P-IOL delivery system has the potential to increase cataract throughput and revenue. This outcome is of interest in a healthcare environment that is increasingly focused on improving efficiency and time savings. The application of P-IOL could help to increase the efficiency of the healthcare system and reduce the burden, dramatically increasing the number of cataract patients.

## Data Availability

The datasets for the analysis of the current study are readily available from the corresponding author upon reasonable request.

## Abbreviations

IOL: Intraocular lens
M-IOL: Manually loaded intraocular lens
1-OR: One operating room
P-IOL: Preloaded intraocular lens
2-OR: Two-operating room
